# The Italian guideline on comprehensive geriatric assessment (CGA) for the older persons: a collaborative work of 25 Italian Scientific Societies and the National Institute of Health

**DOI:** 10.1007/s40520-024-02772-0

**Published:** 2024-05-27

**Authors:** Alberto Pilotto, Pierangelo Lora Aprile, Nicola Veronese, Eleonora Lacorte, Wanda Morganti, Carlo Custodero, Paola Piscopo, Elisa Fabrizi, Francesco Della Gatta, Andrea Merlo, Nicola Vanacore

**Affiliations:** 1 Department of Geriatric Care, Neurology and Rehabilitation, Galliera Hospitals, Genoa, Italy; 2 Department of Interdisciplinary Medicine, “Aldo Moro” University of Bari, Bari, Italy; 3 Italian College of General Practitioners and Primary Care, Florence, Italy; 4 Department of Internal Medicine and Geriatrics, University of Palermo, Palermo, Italy; 5 National Center for Disease Prevention and Health Promotion, Italian National Institute of Health, Rome, Italy; 6 Department of Neuroscience, Mental Health and Sense Organs (NESMOS), Faculty of Medicine and Psychology, Sapienza University of Rome, Rome, Italy; 7 Order of Nursing Professions of Padova, Padua, Italy

**Keywords:** Comprehensive geriatric assessment, Guideline, Systematic review, Meta-analysis, Multidimensional prognostic index

## Abstract

**Background:**

The guideline was promoted by the Italian General Practitioners-Primary Care and Geriatrics Hospital-Community Societies and was carried out involving the National Institute of Health and an Expert Panel including representatives from 25 Scientific and Health-Professional Organizations. The aim of the Guideline was to develop evidence-based recommendations on the efficacy of CGA in older people across different clinical settings and the accuracy and utility of CGA-based tools to assess prognosis.

**Methods:**

According to the methodological handbook of the Italian National System of Guidelines and NICE criteria (National Institute for Health and Care Excellence in England), the Guideline was produced based on the Grading of Recommendations Assessment, Development and Evaluation. Over 20,000 records gathered through databases searches were initially selected. Sixteen recommendations on CGA efficacy were defined based on 117 studies that met the inclusion criteria and were performed in general practices and primary care (26 studies included), medical and surgical clinics (16 studies), emergency departments (17 studies), hospital medical and surgical wards (53 studies), long-term care facilities and nursing homes (5 studies), hospices and palliative care networks (no studies). Nine recommendations on CGA-based prognostic tools were issues based on 42 included studies carried out in general practices and primary care (5 studies), medical and surgical clinics (4 studies), and hospital wards (33 studies).

**Results:**

Using CGA can be useful to reduce hospitalization, mortality, institutionalization, the risk of delirium, and improve appropriateness in drug prescription and maintain functional activities in different settings. Further research on the efficacy of CGA in rehabilitative facilities, nursing homes, and hospice and palliative-care settings is recommended. CGA-based tools, particularly the Multidimensional Prognostic Index, should be used to predict some negative outcomes in different settings.

**Conclusions:**

This Guideline may be useful in clinical practice and as a tool to support research on the use of CGA in older people.

**Supplementary Information:**

The online version contains supplementary material available at 10.1007/s40520-024-02772-0.

## Introduction

Comprehensive Geriatric Assessment (CGA) is usually defined as ”a multidimensional, multidisciplinary process that identifies a person's medical, social, and functional needs and the development of an integrated and coordinated care plan to address these needs” [1]. The main goals of CGA are to improve diagnostic accuracy, optimize medical treatment, improve health outcomes (including functional status and quality of life), optimize the living environment, minimize the use of unnecessary services, and organize a personalized long-term management of older people [2]. Over the years, the application of CGA in different clinical (hospital, home, nursing homes, etc.) and organizational contexts produced a certain degree of heterogeneity in its operational definition. [3]

Despite the heterogeneity of its definition, the dimensions that CGA aims to measure are usually grouped into at least four domains: physical health (e.g., medical history, physical examination, laboratory data, and list of conditions, disease-specific indicators and prevention practices), functional status (e.g., activities of daily living and instrumental activities of daily living [ADLs, IADLs], and other parameters such as mobility and quality of life), psychological health (e.g., cognitive and affective status), and socio-environmental parameters (social network, support needs, safety and adequacy of the living environment). [1]

One of the most innovative aspects that has recently emerged in relation to CGA is its role in predicting the prognosis of older people.[4]. The multidimensional parameters explored by CGA, such as functional status, multimorbidity and socio-economic determinants, are among the most relevant prognostic indicators of negative outcomes in older people, such as mortality [5]. However, only few of the prognostic tools reported in literature have a multidimensional construct based on a CGA [6]. The value of prognosis in older people is becoming increasingly relevant as prognostic definitions can be useful to facilitate some clinical decisions [5].

Therefore, it is important to both identify methodologically validated published multidimensional prognostic tools based on CGA, and to promote a culture of the use of patient prognosis in order to facilitate discussion between healthcare professionals and patients (and/or their caregivers) on shared clinical decisions based on scientific evidence.

Furthermore, CGA can be applied across different care settings (e.g., primary care, hospital, local health and social care facilities), with different types of pathways and levels of intensity. Unfortunately, there are some barriers to the implementation of CGA, such as: 1) an inadequate definition of the characteristics of patients who could benefit the most from a multidimensional care pathway [1]; 2) a high heterogeneity of operational definitions of CGA [7]; 3) a lack specific training on CGA for social and healthcare staff involved in caring for older people [8]; 4) a poor definition of the professional skills required in the interdisciplinary team for drafting, implementing and monitoring CGA-based treatment plans. [9]

Despite it being widely used for more than three decades [3] and several studies being published on its relevance and efficacy across different settings and conditions [10], to our knowledge, no clinical guidelines on CGA are currently available.

Therefore, the main aim of this guideline was to produce, based on the analysis of currently available scientific literature, clinical and research recommendations on: 1) the efficacy of CGA interventions in improving a series of outcomes, 2) the accuracy and utility of multidimensional tools in predicting outcomes in older people.

## Materials and methods

### Expert panel

This guideline, promoted by the Società Italiana Geriatria Ospedale e Territorio (SIGOT) and the Società Italiana Medicina Generale e Cure Primarie (SIMG), included a total of 25 societies and stakeholders. Methodological support was provided, throughout all the activities, by the Istituto Superiore di Sanità (ISS). The working group included healthcare professionals, representatives of the main national scientific societies, involved in the care and management of older people, i.e. geriatricians, cardiologists, nephrologists, neurologists, oncologists, psychiatrists, prevention medicine specialists, physiatrists, geriatric surgeons, orthopedists, urologists, emergency physicians, palliative care physicians. The panel of experts included general practitioners, geriatricians and specialists in other medical and surgical areas, epidemiologists, nurses, social workers, psychologists, occupational and physical therapists, speech and language therapists, statisticians, and experts in health economics and in bioethics. The panel also included patients' representatives to ensure consideration of their values, priorities, and preferences. The whole panel's opinion was also essential to identify welfare and organizational problems related to CGA. In the Acknowledgment section, we reported the list of all the societies and stakeholders involved.

This guideline was developed following the methodological handbook for the production of clinical practice guidelines developed by the National Center for Clinical Excellence, Quality and Safety of Care of the Italian National Institute of Health (Centro Nazionale per l’Eccellenza Clinica, la Qualità e la Sicurezza delle Cure dell’Istituto Superiore di Sanità—version 1.3.3, last update March 2023). Its core methodology is based on the Grading of Recommendations Assessment, Development and Evaluation (GRADE) [11]. To assess the certainty of evidence from studies on multidimensional prognostic tools, we used an approach similar to the one proposed by the National Institute for Health and Clinical Excellence (NICE) that has several domains in common with GRADE [12]. The Evidence to Decision Framework was used to inform the process from the GRADE summary of evidence to recommendations [13]. Finally, to improve the quality of the guidelines, two external expert referees assessed the methodology, content and recommendations, providing comments that were considered by panel members in the final version of the guidelines.

## Review questions and search strategy

The definition of the review questions was based in the identification of their relative PICOs (Patient or Population, Intervention, Comparison and Outcome) according to setting. After the identification of PICOs, the panel of experts voted the outcomes of interest based on their clinical expertise.

Based on the PICOs questions, structured searches were performed on the scientific databases Cochrane Library, PubMed and Embase, using the following keywords ('geriatric assessment', 'comprehensive geriatric assessment', 'multidimensional geriatric assessment') adapted to nine clinical settings categorized in 5 sections: Sect. 1. Outpatients in specialist clinics and primary care/general medicine/community: setting 1) general practice and primary care; setting 2) specialist medical outpatient clinics; setting 3) specialist surgical outpatient clinics; Sect. 2. Patients in emergency departments: setting 4) emergency department. Section 3. Patients admitted to hospital: setting 5) hospital medical wards; setting 6) hospital surgical wards; Sect. 4. Patients in long-term care facilities: setting 7) long-term rehabilitation facilities; setting 8) nursing homes; Sect. 5. Patients in hospice and other palliative care facilities: setting 9) Hospice and other palliative care facilities. Bibliographic searches were run from database inception to 19th November 2022 for all topics.

## Study selection

Identified records were screened by two independent reviewers for each review question, using Rayyan (https://www.rayyan.ai/) in a two-step approach, with an initial screening based on titles and abstracts followed by a second step in which full texts of the studies identified were applied predefined eligibility criteria. Any conflicts were resolved by at least one member of the expert panel. Two types of studies were selected: 1. Intervention studies: randomized controlled trials (RCTs) and pre/post studies, 2. Prognostic studies: prospective and retrospective studies reporting tools using a multidimensional pathway as exposure and reporting the association with the outcomes of interest in terms of accuracy (Area Under the Curve, AUC) or precision (C-index, Brier Index, pseudoR2) with their 95% confidence intervals (CIs). The AUC is the most commonly used metric for assessing the accuracy of predictive tools and compare it across different tools[14], while C-index is the most commonly used metric for precision.

## Data extraction

For each review question, data from included studies were extracted by two members of the Evidence Review Team (ERT), and subsequently revised by another independent ERT member, using a structured Microsoft Excel spreadsheet. For each eligible study, the following data were extracted: author, year, type of study (RCT, pre/post, prospective or retrospective), setting, condition, presence and type of comorbidities, number of participants, mean age, percentage of females, length of follow-up (months), number of domains considered in the multidimensional evaluation, outcomes of interest. Information for quality assessment was also extracted.

## Quality assessment

We used the Cochrane Collaboration’s tool for assessing risk of bias in randomised trials (RoB) for RCTs [15] and the Newcastle–Ottawa Scale (NOS) for quality assessment of non-randomized studies (pre/post, retrospective and prospective). [16]

## Evaluation of the quality of evidence and formulation of recommendations

Evidence from meta-analyses was evaluated using the GRADE assessment. The GRADE framework includes several relevant domains to assess the certainty of evidence, including study design, risk of bias, inconsistency, indirectness, imprecision and other aspects, such as publication bias [17].

The certainty of evidence was classified as very low (the true effect is probably significantly different from the estimated effect), low (the true effect might be significantly different from the estimated effect), moderate (the true effect is probably close to the estimated effect) or high (the true effect is probably similar to the estimated effect) [17]. Data from literature were analyzed using the GRADEpro Guideline Development Tool (McMaster University, 2015; developed by Evidence Prime, Inc.). “Evidence-based Recommendations” were produced based on the GRADE methodology. The direction, strength and wording of the recommendations were determined according to the GRADE evidence profiles. The certainty of evidence was defined according to the GRADE from very low to high; the strength of recommendations (strong or weak) was based on the Evidence to Decision Framework. [18]

According to the GRADE methodology, the strength of the recommendations was attributed based on the following elements:

1. Relevance of the considered topic and of the considered outcomes;
2. Balance between desirable and undesirable outcomes (trade-off) taking into account the best estimates of the size of both desirable and undesirable effects and the relevance of considered outcomes;
3. Overall quality of the body of evidence;
4. Impact on the use of resources;
5. Equity, acceptability and feasibility;

## Results

### Systematic literature review

Database searches led to identifying 26.130 records, 117 of these studies met the inclusion criteria for intervention studies and 42 for prognostic studies (See Table 1). When considering PICOs, eight outcomes were identified shared among the 9 settings: mortality rates, impact on functional status and on quality of life, appropriateness of drug prescription and number of drugs, rates of admission to emergency department, hospital wards and/or long-term care facilities. Other specific outcomes were investigated in different settings (*e.g.,* inclusion into a palliative care network).

**Table 1:** Numbers of records retrieved, screened, and excluded for both intervention studies and prognosis studies, divided by each setting

	Primary care and general pratice	Outpatients–medical area	Outpatients–surgical area	Emergency department	Hospital–medical area	Hospital surgical area	Long term care rehabilitative facilities	Nursing home	Pallative care	Total
Records retrived	3.948	988	2.675	2.211	7.741	3.343	2.807	1.080	1.337	26.130
Duplicate records removed	1.054	156	624	139	2318	1164	10	308	246	6.019
Records screened	2894	832	2.051	2.072	5.423	2.179	2.797	772	1.091	20.111
Recorde excluded	2.789	772	1.964	1.966	5.274	2.123	2.768	755	1.085	19.496
Eligible studies	105	60	87	106	149	56	29	17	6	615
studies Included–Intervention studies	26	11	5	17	37	16	4	1	0	117
studies Included–Prognosis studies	5	3	1	0	33	0	0	0	0	42

Most of the studies included in the systematic review enrolled hospitalized older people, accounting for almost 38% of the overall screened records (7.602/20.111), followed by studies enrolling people in the primary care and general practice settings, accounting for 14.4% of the overall screened records (2.894/20.111).

## Findings from intervention studies

Results from the included interventional studies carried-out in primary care and general practice setting showed that people allocated on CGA had a lower rate of hospitalization compared to usual care (17% lower). Outpatients in specialist clinics (both medical and surgical areas) allocated to CGA also had lower mortality rates (12% lower), incidence of delirium (56% lower), and length of hospital stay (2-day shorter) compared to outpatients on usual care, and had a higher drug prescription appropriateness (in oncological patients who underwent chemotherapy). People in emergency department allocated to CGA showed lower hospitalization rates (9% lower), functional impairment (24% lower), and rate of re-admission to emergency care (11% lower) compared to usual care. Hospitalized patients in both medical and surgical wards allocated to CGA showed a significantly lower rate of admission to long-term care facilities (13% lower) and incidence of delirium (24% lower).

**Fig. 1:**
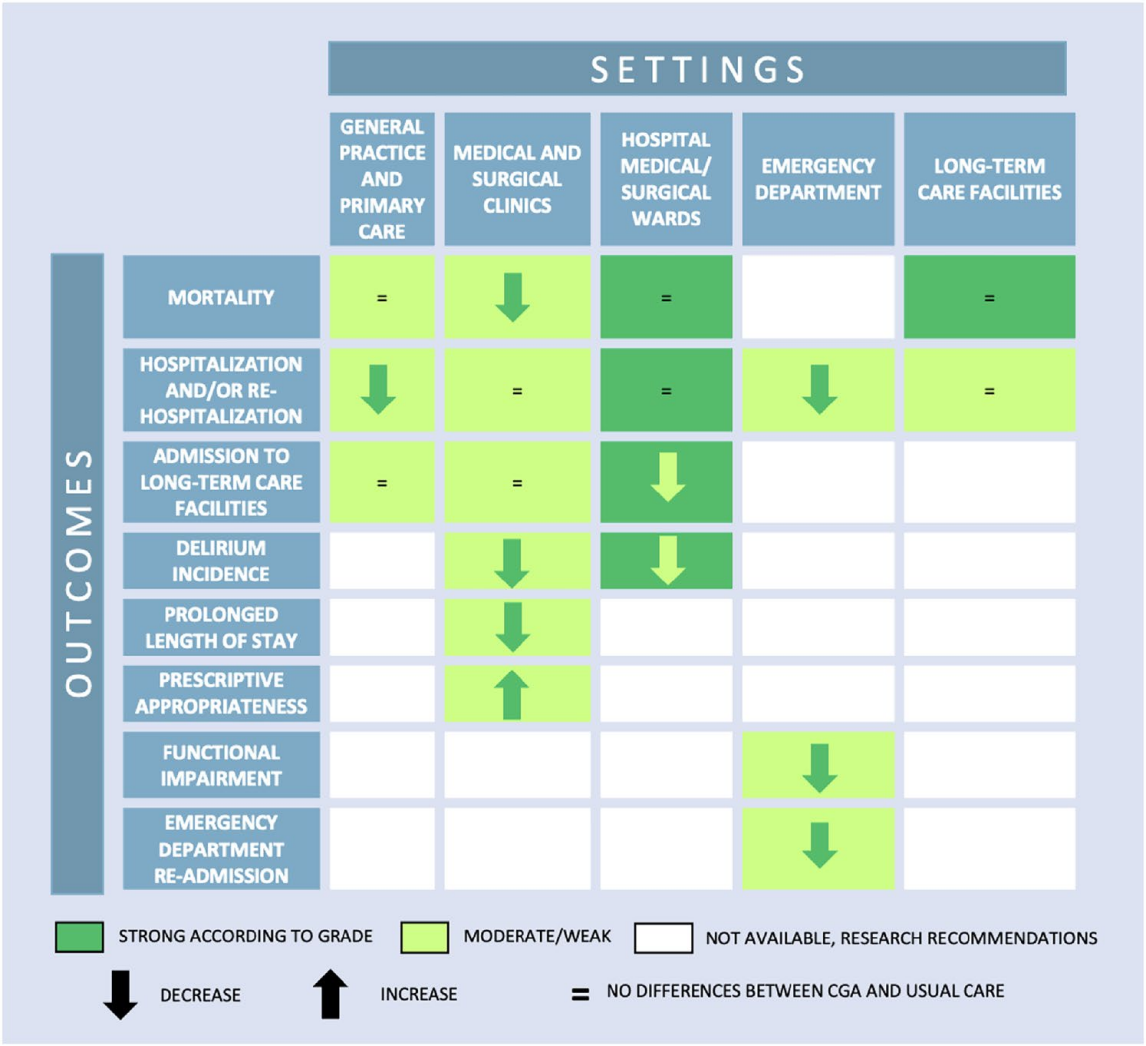
Efficacy of CGA compared to usual care in Randomized Clinical Trials across different settings

No differences between CGA and usual care were observed in hospitalization and mortality rates in people in long-term care facilities. No intervention studies were retrieved investigating CGA in the hospice and other palliative care network setting, and therefore no clinical practice recommendations could be produced for this setting. For all outcomes where no data were available from literature across different settings (see the white cells in Fig. 1), a series of research recommendations were defined by the expert group (see below).

The main findings of the intervention studies are graphically reported in Fig. 1.

## Findings from prognostic studies

No eligible prognostic studies were identified for five out of the nine considered settings. Specifically, no studies were retrieved for emergency department, hospital surgical wards, long-term care facilities (both rehabilitative structures and nursing homes), and hospice and palliative care network.

The main considered outcome for primary care and general practice was mortality. The investigated tools in literature were the Multidimensional Prognostic Index (MPI), the Multidimensional Geriatric Prognostic Index (MGPI), and the Resident Assessment Instrument for Home Care (RAI- HC).

The MPI tools include seven to nine domains with 27– 55 items, the MGPI included 8 domains with 104 items, while the RAI-HC is structured in 15 domains with 400 items (See Table 2). Overall, the MPI appeared to report a higher precision (C-Index) in predicting mortality (after one month, one year, 10 years, and 15 years from the assessment) (See Table 2). Data on accuracy (AUC) were available only for two studies on MPI reporting an overall very good accuracy of this tool (AUC values between 0.80 and 0.90).

**Table 2:** Precision (C-Index) and accuracy (AUC) of CGA-based tools to predict mortality in older adults referring to primary care and general practice (setting 1). References of the articles in Table are reported in Appendix A in the supplementary material

CGA-based tools	Number of domains (Number of items)	Sample size	Follow-up	C-Index (95% CI)	Degree of certainty	AUC (95% CI)	Degree of certainty
MPI-SvaMA Development cohort	9 (55)	7.876	1 month	0.83 (0.82–0.84)	Moderate		
MPI-SvaMA Validation cohort	9 (55)	4.144	1 month	0.83 (0.82–0.85)	Moderate		
RAI- HC	15 (400)	435.009	6 months	0.75 (0.75–0.76)	Moderate		
MPI-SvaMA Development cohort	9 (55)	7.876	1 year	0.79 (0.78–0.80)	Moderate		
MPI-SvaMA Validation cohort	9 (55)	4.144	1 year	0.79 (0.78–0.80)	Moderate		
Multidimensional Geriatric Prognostic Index Development cohort	8 (105)	988	5 years	0.80 (0.76–0.83)	Moderate		
Multidimensional Geriatric Prognostic Index Validation cohort	8 (104)	1.109	5 years	0.80 (0.77–0.82)	Moderate		
MPI-ELSA	7 (27)	6.244	10 years	0.81 (0.80–0.82)	Moderate	0.80	Very Low
MPI-InChianti	8 (72)	1.453	15 years	0.82 (0.81–0.84)	Moderate	0.86 (0.84 – 0.88)	Moderate

*AUC *area under the curve*; CI *confidence intervals*; MPI *Multidimensional Prognostic Index*; RAI-HC *Resident Assessment Instrument for Home Care*; ELSA *English Longitudinal Study on Ageing

In outpatient in both in medical and surgical specialist clinics, MPI was reported as the most accurate CGA-based prognostic tool in predicting mortality and post-operative complications.

A higher number of studies were retrieved reporting data on people hospitalized in medical wards (see Table 3). Therefore, five different outcomes were considered: mortality, admission to long-term care facilities, re-hospitalization, length of hospital stay (LOS), and incidence of delirium. The MPI showed good and very good accuracy and precision in predicting mortality at 1 month (AUC = 0.79; 95% CI = 0.76–0.81; C Index = 0.82; 95%CI = 0.78–0.85), 6 months (AUC = 0.74; 95% CI = 0.71–0.76; C Index = 0.79; 95% CI = 0.73–0.85), and 12 months (AUC = 0.72; 95% CI = 0.69–0.76; C Index = 0.76; 95% CI = 0.71–0.81). The CGA-based predictive score was reported to be accurate in predicting mortality at 12 months (AUC = 0.73; 95% CI = 0.73–0.74), while the Mortality Risk Index (MRI) and the Hospitalized Older Patient Examination Index (HOPE Index) were reported as able to predict mortality at 24 months with an accuracy of 0.72 (0.69 –0.75) and 0.68 (0.63–0.73), respectively. The HOPE Index showed moderate accuracy in predicting re-hospitalization 24 months after hospital discharge (AUC = 0.61; 95% CI = 0.58 –0.64).

**Table 3:** Accuracy of CGA-based tools to predict outcomes in hospitalized older people (setting 5). References of the articles in Table are reported in Appendix B in the supplementary material

Outcomes	Tool	Number of studies	Sample size	Follow-up	Risk of bias	Inconsistency	Lack of results’ reproducibility	Inaccuracy	AUC	Lower 95%	Upper 95%	Quality
In-hospital Mortality	CGA-derived score	1	581	Length of hospital stay	severe	Not present	Severe	Severe	0.86	0.79	0.92	Low
Mortality (< 1 month)	MPI	13	11.787	1 month	Not present	present	Not present	Not present	0.79	0.76	0.81	Moderate
Mortality (6 months)	MPI	12	9.751	6 months	Not present	present	Not present	Not present	0.74	0.71	0.76	Moderate
Mortality (12 months)	CGA-based Predictive Score	1	3.112	12 months	Not present	Not present	severe	Not present	0.73	0.73	0.74	Moderate
Mortality (12 months)	FI-CGA	3	2.568	12 months	Not present	very high	Not present	Not present	0.71	0.61	0.81	Low
Mortality (12 months)	MPI	14	13.997	12 months	Not present	present	Not present	Not present	0.72	0.69	0.76	Moderate
Mortality (24 months)	HOPE index	1 (two cohorts)	3.043	24 months	Not present	Not present	Severe	Not present	0.68	0.63	0.73	Moderate
Mortality (24 months)	MRI	1 (two cohorts)	1.306	24 months	Not present	Not present	Severe	Not present	0.72	0.69	0.75	Moderate
Admission rate to long-term care facilities	MPI	1	1.140	12 months	not present	not present	Severe	not present	0.81	0.78	0.85	Moderate
Re-hospitalization rate (12 months)	FI-CGA	2	535	12 months	Severe	Very high	Not present	Not present	0.61	0.53	0.68	Very Low
Re-hospitalization rate (12 months)	MPI	2	2.834	12 months	Not present	Very high	Not present	Not present	0.70	0.63	0.77	Low
Re-hospitalization rate (24 months)	HOPE index	1 (two cohorts)	3.043	24 months	Not present	Not present	Severe	Not present	0.61	0.58	0.64	High
Prolonged length of Hospital Stay	MPI	1	1.467	1	Not present	Not present	Severe	Not present	0.57	0.54	0.60	Moderate
Prolonged length of Hospital Stay	MPI	1	1.190	12	Not present	Not present	Severe	Not present	0.57	0.53	0.60	Moderate
Delirium incidence	MPI	1	697	Length of hospital stay	Not present	Not present	Severe	Not present	0.63	0.57	0.70	Moderate

*AUC *area under the curve*; FI-CGA *frailty index based on a comprehensive geriatric assessment*; HOPE *hospitalized older patient examination*; MPI *multidimensional prognostic index*; MRI *mortality risk index

Finally, the MPI showed very good accuracy in predicting admission to long-term care facilities (AUC = 0.81; 95% CI = 0.78–0.85), and low–moderate accuracy to predict the risk of prolonged LOS (AUC = 0.57; 95% CI = 0.53–0.60) with good precision (C Index = 0.74: 95% CI = 0.71–0.76), and the onset of delirium (AUC = 0.63; 95% CI = 0.57–0.70) in older people admitted to hospital (Table 3).

## Clinical recommendations

Based on the analysis of data from literature and discussion of the EtD framework, 25 clinical practice recommendations were developed and approved by the expert panel (See Table 4).

**Table 4:** Recommendations about comprehensive geriatric assessment

#	Recommendation	Strength of recommendation
R1a.1	Using comprehensive geriatric assessment is suggested to reduce hospitalization rate in older people referred to general practice and primary care	Weak in favor
R1a.2	Using comprehensive geriatric assessment is not suggested for the sole purpose of decreasing mortality rate or institutionalization in older people referred to general practice and primary care clinics	Weak in against
R1b.1	Using comprehensive geriatric assessment, through the multidimensional prognostic index (MPI) and the Resident Assessment Instrument for Home Care (RAI-HC), is suggested to predict the short-, medium- and long-term risk of death in older people referred to general practice and primary care clinics	Weak in favor
R2a.1	Using comprehensive geriatric assessment is suggested to reduce mortality rate in older people referred to specialist medical clinics	Weak in favor
R2a.2	Using comprehensive geriatric assessment is not suggested for the sole purpose of reducing hospitalization and institutionalization rate in older people referred to specialist medical clinics	Weak in against
R2a.3	Using comprehensive geriatric assessment is suggested to increase the appropriateness of prescribing (discontinuation of chemotherapy due to toxic effects) in older people referred to specialist medical clinics	Weak in favor
R2b.1	Using comprehensive geriatric assessment, through the multidimensional prognostic index, is suggested to predict the risk of death in older people with cancer and referred to specialist clinics in the medical area	Weak in favor
R3a.1	Using comprehensive geriatric assessment is suggested to reduce the length of stay in older people referred to specialist surgical outpatient clinics	Weak in favor
R3a.2	Using comprehensive geriatric assessment is suggested to reduce the incidence of post-operative delirium in older people referred to specialist surgical outpatient clinics	Weak in favor
R3b.1	Using comprehensive geriatric assessment, through the multidimensional prognostic index, is suggested to predict post-operative complications in older people with colorectal cancer referred to specialist surgical outpatient clinics	Weak in favor
R4a.1	Using comprehensive geriatric assessment is suggested to reduce hospitalization rate in older people admitted to the emergency department	Weak in favor
R4a.2	Using comprehensive geriatric assessment is suggested to reduce functional impairment, over a period of 4 to 12 months, in older people admitted to the emergency department	Weak in favor
R4a.3	Using comprehensive geriatric assessment is suggested to reduce readmission rates to the emergency department within two weeks to 12 months after the first access, in older people admitted to the emergency department	Weak in favor
R5a.1	Using comprehensive geriatric assessment is recommended to reduce institutionalization rate in older people admitted to hospital medical wards	Strong in favor
R5a.2	Using comprehensive geriatric assessment is not recommended for the sole purpose of reducing mortality or re-hospitalization rate in older people admitted to hospital medical wards	Strong in against
R5b.1	Using comprehensive geriatric assessment, through the multidimensional prognostic index (MPI), is suggested to predict the risk of short- (< 1 month), medium- (6 months) and long-term (12 months) mortality in older people admitted to hospital medical wards	Weak in favor
R5b.2	Using comprehensive geriatric assessment, through a CGA-based Predictive Score (DAMAGE study), is suggested to predict the risk of death at 12 months, and, through the HOPE Index (Hospitalized Older Patient Examination) and the Mortality Risk Index (MRI) at 24 months, in older people admitted to hospital medical wards	Weak in favor
R5b.3	Using comprehensive geriatric assessment, through the multidimensional prognostic index (MPI), is suggested to predict the risk of institutionalization in older people admitted to hospital medical wards	Weak in favor
R5b.4	Using comprehensive geriatric assessment, through the HOPE Index, is recommended to predict the risk of re-hospitalization at 24 months in older people admitted to hospital medical wards	Strong in favor
R5b.5	Using comprehensive geriatric assessment, through the multidimensional prognostic index (MPI), is suggested to predict the risk of prolonged hospital stay in older people admitted to hospital medical wards	Weak in favor
R5b.6	Using comprehensive geriatric assessment, through the multidimensional prognostic index (MPI), is suggested to identify older people admitted to hospital medical wards that are at a higher risk of delirium	Weak in favor
R6a.1	Using comprehensive geriatric assessment is recommended to reduce the incidence of delirium in older people admitted to orthogeriatric wards and referred to surgical wards	Strong in favor
R6a.2	Using comprehensive geriatric assessment is not recommended for the sole purpose of reducing mortality or institutionalization rates in nursing homes or re-hospitalization rate in older people admitted to orthogeriatric and in surgical hospital wards	Strong in against
R7a.1	Using comprehensive geriatric assessment is not recommended for the sole purpose of reducing mortality rate in older people admitted to long-term care rehabilitation facilities	Strong in against
R8a.1	Using comprehensive geriatric assessment is not suggested for the sole purpose of reducing mortality or hospitalization rates in older people resident in nursing homes	Weak in against

## Research recommendations

Acknowledging the limitations of the evidence available on some specific topics, the expert panel underlined the need to improve the quality of research to further clarify the potential utility of CGA in:

- Improving functional status and quality of life (in primary care, medical and surgical clinics and wards, long-term care rehabilitation facilities and nursing homes, hospice and palliative care networks)
- Improving the early identification of palliative care needs
- Reducing mortality (in outpatient care of surgical area, emergency department and nursing homes)
- Reducing hospitalization (from nursing homes, hospice and palliative care network)
- Reducing access rate to emergency department (in primary care setting and after discharge from the hospital medical wards)
- Reducing length of hospital stay (in surgical hospital setting)
- Optimizing post-discharge management pathways (in medical and surgical hospital setting)
- Improving medications appropriateness (in primary care, medical hospital setting and nursing homes)
- Reducing post-operative complications (in surgical outpatient and hospital settings)
- Minimizing use of physical restraints (in medical hospital setting and nursing homes)

The expert panel also underlined the lack of CGA-based prognostic tools able to predict negative outcomes in patients admitted to the emergency department, in people hospitalized in surgical wards or resident in long term-care rehabilitation facilities and nursing homes, or in hospices or palliative care networks. Therefore, recommendations for further research were included to cover these issues.

## Discussion

This Guideline was produced adopting a multidisciplinary view, involving some of the most relevant Italian scientific societies working in the field of geriatrics, general and internal medicine, and along with medical and non-medical societies and stakeholders interested in this topic.

The included recommendations support providing individualized care to older people, and prioritizing care through the identification of individuals at increased risk of negative health outcomes. Moreover, this Guideline provides indications on the most relevant multidimensional tools that are available for use by healthcare and social professionals involved in caring older people across different settings and contexts. At the same time, not all aspects related to the complex care of older people could be covered in this Guideline, due to the limited available evidence, and recommendations could not be issued for all the predefined review questions. However, in these cases indications for future research were provided.

## Implementation, updating and dissemination

Considering the continuous evolution of medical and scientific knowledge, the document is to be updated within 3 years (January 2026).

Multiple ways of disseminating the document will be adopted, including the following approaches:

- Dissemination in media and press;
- Mail shipment to reference centers;
- Publication on the SNLG-ISS website and on the websites of scientific societies, health agencies, etc.;
- Scientific publications;
- Presentation at national and international conferences.

When considering factors facilitating the clinical and organizational application of this Guideline, scientific literature suggests the following as relevant factors:

- Developing training and educational programs specifically including information on the utility and operational methods of CGA. This Guideline suggests, based on scientific literature, multidimensional tools and their most appropriate and useful application for patients, caregivers, and health and social care professionals. To this purpose, training courses can be proposed, both in presence and online, integrated with practical exercises involving the application of real and/or simulated clinical case studies taken from clinical practice;
- Promoting at local level of CGA paths integrated into specific pathways compatible with individual territorial realities;
- Widening the communication skills of the health and social care professionals included as target population in the Guideline to facilitate the implementation of multidimensional assessment paths and the planning of individualized care programs.

Among the possible obstacles to the dissemination and implementation of this Guideline, the need for a digital computerization of these tools should be mentioned to overcome the current heterogeneity of multidimensional tools, and to facilitate the sharing of information and shorten the CGA procedures. The need for organizational adaptations should also be considered, including the acquisition of trained and dedicated personnel in the different settings (hospital, territorial and residential areas) that the application of the recommendations of this Guideline may require.

## Concluding remarks

This Guideline provides recommendations on the implementation of a multidimensional CGA-based approach when caring for older people in different clinical settings in order to improve several negative outcomes. Moreover, the Guideline recommends the use of CGA-based prognostic tools as a support to provide the most appropriate and cost-effective management approach, and as a facilitating tool for decision making for each individual patient. The included research recommendations highlight those clinical topics where further studies are needed to identify where and when a multidimensional approach could be useful to support patients, caregivers, physicians, and health and social care professionals.

## Working group on the italian guideline on cga for the older persons

### Panel of experts

Chiara Amendola, RSA Residenza Bellagio (CO).

Andrea Arighi, IRCCS Ca' Granda Ospedale Maggiore Policlinico, Milano.

Rodolfo Brianti, Azienda Ospedaliero‐Universitaria di Parma, Parma.

Antonella Brunello, Istituto Oncologico Veneto, Padova.

Emanuele Caroppo, ASL Roma 2, Roma.

Luca Carmignani, IRCCS Policlinico San Donato, Milano.

Alberto Castagna, Azienda Sanitaria Provinciale di Catanzaro, Catanzaro.

Matteo Cesari, Università di Milano, Milano.

Andrea Fabbri, AUSL Romagna, Forlì.

Valeria Fava, stakeholder expert.

Roberto Gatti, Humanitas University, Milano.

Fabrizio Giunco, IRCCS Fondazione Don Carlo Gnocchi, Milano.

Ignazio Grattagliano, Monopoli, Bari.

Francesco Malci, Università degli Studi di “Tor Vergata”, Roma.

Mara Meneghel, Centro Servizi per Anziani Bonora, Camposampiero, Padova.

Andrea Merlo, Ordine delle Professioni Infermieristiche, Padova.

Piergiorgio Messa, Università degli Studi di Milano, Milano.

Mara Morini, Società Italiana Igiene, Bologna.

Enrico Mossello, Università degli Studi di Firenze, Firenze.

Elisabetta Neve, Università degli Studi di Verona, Verona.

Alessandro Padovani, Università degli Studi di Brescia, Brescia.

Alessandro Puzziello, Università degli Studi di Salerno, Salerno.

Emilio Romanini, Polo Sanitario San Feliciano, Roma.

Renzo Rozzini, Fondazione Poliambulanza – Istituto Ospedaliero, Brescia.

Marco Tinelli, Centro Auxologico di Milano, Milano.

## Evidence review team

Margherita Azzini, ULSS 9 Scaligera Regione Veneto, Verona.

Guido Bellomo, Istituto Superiore di Sanità, Roma.

Virginia Boccardi, Università degli Studi di Perugia, Perugia.

Enrico Brunetti, A.O.U. Città della Salute e della Scienza, Torino.

Alberto Cella, ASL 2 Regione Liguria, Savona.

Stefano Celotto, Udine.

Carlo Custodero, Università degli Studi di Bari, Bari.

Jacopo Demurtas, Capalbio, Grosseto.

Francesco Della Gatta, Università La Sapienza, Roma.

Elisa Fabrizi, Istituto Superiore di Sanità, Roma.

Lucia Muraca, Catanzaro.

Giulio Nati, Roma.

Claudia Piccioni, Aosta.

Alessandra Ceccarini, Istituto Superiore di Sanità, Roma.

## Bioethics expert:

Luciana Riva, Istituto Superiore di Sanità, Roma.

## Economic analysis team:

Francesco Saverio Mennini, Università “Tor Vergata”, Roma.

Paolo Sciattella, Università “Tor Vergata”, Roma.

Statement of human rights: Not needed.

Animal rights: Not needed.

Informed consent statement: Not needed.

### Supplementary Information

Below is the link to the electronic supplementary material.

Supplementary file1 (DOCX 21 KB)

## Data Availability

The full text of the Guideline on Comprehensive Geriatric Assessment for Older Person is available in Italian language at the following link: https://www.iss.it/-/valutazione-multidimensional-persona-anziana-1.
