# Supplementary material for: The Italian guideline on comprehensive geriatric assessment (CGA) for the older persons: a collaborative work of 25 Italian Scientific Societies and the National Institute of Health
Source: Aging Clin Exp Res. 2024 May 27;36(1):121. doi: 10.1007/s40520-024-02772-0 (PMC11128394; doi:10.1007/s40520-024-02772-0)
Supplement: Supplementary file 1 — Supplementary file1 (DOCX 21 KB) [file 40520_2024_2772_MOESM1_ESM.docx]

**Appendix A – References Table 2**

Hsu AT, Manuel DG, Spruin S, Bennett C, Taljaard M, Beach S, Sequeira Y, Talarico R, Chalifoux M, Kobewka D, Costa AP, Bronskill SE, Tanuseputro P (2021) Predicting death in home care users: derivation and validation of the Risk Evaluation for Support: Predictions for Elder-Life in the Community Tool (RESPECT). CMAJ, 193(26), E997-E1005.

Jung HW, Kim JW, Han JW, Kim K, Kim JH, Kim KI, Kim CH, Kim KW (2016) Multidimensional Geriatric Prognostic Index, Based on a Geriatric Assessment, for Long- Term Survival in Older Adults in Korea. PloS one, 11(1), e0147032.

Pilotto A, Gallina P, Fontana A, Sancarlo D, Bazzano S, Copetti M, Maggi S, Paroni G, Marcato F, Pellegrini F, Donato D, Ferrucci L.(2013) Development and validation of a Multidimensional Prognostic Index for mortality based on a standardized Multidimensional Assessment Schedule (MPI-SVaMA) in community-dwelling older subjects. J Am Med Dir Assoc, 14(4), 287-292.

Pilotto A, Veronese N, Siri G, Bandinelli S, Tanaka T, Cella A, Ferrucci L (2021) Association Between the Multidimensional Prognostic Index and Mortality During 15 Years of Follow-up in the InCHIANTI Study. J Gerontol A Biol Sci Med Sci, 76(9), 1678-1685.

Veronese N, Noale M, Cella A, Custodero C, Smith L, Barbagelata M, Maggi S, Barbagallo M, Sabbà C, Ferrucci L, Pilotto A (2022) Multidimensional frailty and quality of life: data from the English Longitudinal Study of Ageing. Qual Life Res, 31(10), 2985-2993.

**Appendix B – References Table 3**

Abbatecola AM, Spazzafumo L, Corsonello A, Sirolla C, Bustacchini S, Guffanti E (2011) Development and validation of the HOPE prognostic index on 24-month posthospital mortality and rehospitalization: Italian National Research Center on Aging (INRCA). Rejuvenation Res, 14(6), 605-613.

Bartoli G, Omiciuolo C, Fiorenzato F, Russi E, Ceschia G, Maglione M, Bevilacqua L (2019) Oral health status as predictor of 1-year mortality after discharge from an acute geriatric unit. European geriatric medicine, 10(6), 889-897.

Bryant K, Sorich MJ, Woodman RJ, Mangoni AA (2019) Validation and adaptation of the multidimensional prognostic index in an older Australian cohort. Journal of clinical medicine, 8(11), 1820.

Carriere C, Stolfo D, Baglio V, Gerloni R, Merlo M, Barbati G, Cannata A, Biolo G, Sinagra G (2018) Outcome of the multidimensional prognostic index in ultra-octogenarian patients hospitalized for cardiovascular diseases. J Cardiovasc Med (Hagerstown), 19(10), 536-545

De Luca E, Perissinotto E, Fabris L, Pengo V, Zurlo A, De Toni P, De Zaiacomo F, Manzato E, Giantin V (2015) Short- and longer-term predictive capacity of the Multidimensional Prognostic Index: The timing of the assessment is of no consequence. Arch Gerontol Geriatr, 61(3), 458-463.

Deschasse G, Bloch F, Drumez E, Charpentier A, Visade F, Delecluse C, Loggia G, Lescure P, Attier-Zmudka J, Bloch J, Gaxatte C, Van Den Berghe W, Puisieux F, Beuscart JB (2022) Development of a Predictive Score for Mortality at 3 and 12 Months After Discharge From an Acute Geriatric Unit as a Trigger for Advanced Care Planning. J Gerontol A Biol Sci Med Sci, 77(8), 1665-1672.

Drame M, Novella JL, Lang PO, Somme D, Jovenin N, Laniece I, Couturier P, Heitz D, Gauvain JB, Voisin T, De Wazieres B, Gonthier R, Ankri J, Jeandel C, Saint-Jean O, Blanchard F, Jolly D (2008) Derivation and validation of a mortality-risk index from a cohort of frail elderly patients hospitalised in medical wards via emergencies: the SAFES study. Eur J Epidemiol, 23(12), 783-791.

Gregersen M, Hansen TK, Jorgensen BB, Damsgaard EM (2020) Frailty is associated with hospital readmission in geriatric patients: a prognostic study. Eur Geriatr Med, 11(5), 783-792.

Hansen TK, Shahla S, Damsgaard EM, Bossen SRL, Bruun JM, Gregersen M (2021) Mortality and readmission risk can be predicted by the record-based Multidimensional Prognostic Index: a cohort study of medical inpatients older than 75 years. Eur Geriatr Med, 12(2), 253-261.

Jung HW, Kim JW, Han JW, Kim K, Kim JH, Kim KI, Kim CH, Kim KW (2016) Multidimensional Geriatric Prognostic Index, Based on a Geriatric Assessment, for Long- Term Survival in Older Adults in Korea. PloS one, 11(1), e0147032.

Meyer AM, Pickert L, Heess A, Becker I, Kurschat C, Bartram MP, Benzing T, Polidori MC (2022) Prognostic Signature of Chronic Kidney Disease in Advanced Age: Secondary Analysis from the InGAH Study with One-Year Follow-Up. Biomolecules, 12(3):423.

Noetzel N, Meyer AM, Siri G, Pickert L, Heess A, Verleysdonk J, Benzing T, Pilotto A, Barbe AG, Polidori MC (2021) The impact of oral health on prognosis of older multimorbid inpatients: the 6-month follow up MPI oral health study (MPIOH). Eur Geriatr Med, 12(2), 263-273.

Pilotto A, Addante F, Ferrucci L, Leandro G, D'Onofrio G, Corritore M, Niro V, Scarcelli C, Dallapiccola B, Franceschi M (2009) The multidimensional prognostic index predicts short- and long-term mortality in hospitalized geriatric patients with pneumonia. J Gerontol A Biol Sci Med Sci, 64(8), 880-887.

Pilotto A, Azzini M, Cella A, Cenderello G, Castagna A, Pilotto A, Custureri R, Dini S, Farinella ST, Ruotolo G, Padovani A, Custodero C, Veronese N (2021) The multidimensional prognostic index (MPI) for the prognostic stratification of older inpatients with COVID-19: A multicenter prospective observational cohort study. Arch Gerontol Geriatr, 95, 104415.

Pilotto A, Dini S, Daragjati J, Miolo M, Mion MM, Fontana A, Storto ML, Zaninotto M, Cella A, Carraro P, Addante F, Copetti M, Plebani M (2018) Combined use of the multidimensional prognostic index (MPI) and procalcitonin serum levels in predicting 1-month mortality risk in older patients hospitalized with community-acquired pneumonia (CAP): a prospective study. Aging Clin Exp Res, 30(2), 193-197.

Pilotto A, Ferrucci L, Franceschi M, D'Ambrosio LP, Scarcelli C, Cascavilla L, Paris F, Placentino G, Seripa D, Dallapiccola B, Leandro G (2008) Development and validation of a multidimensional prognostic index for one-year mortality from comprehensive geriatric assessment in hospitalized older patients. Rejuvenation Res, 11(1), 151-161.

Pilotto A, Rengo F, Marchionni N, Sancarlo D, Fontana A, Panza F, Ferrucci L, Group FSS (2012) Comparing the prognostic accuracy for all-cause mortality of frailty instruments: a multicentre 1-year follow-up in hospitalized older patients. PloS one, 7(1), e29090.

Pilotto A, Sancarlo D, Aucella F, Fontana A, Addante F, Copetti M, Panza F, Strippoli GF, Ferrucci L (2012) Addition of the multidimensional prognostic index to the estimated glomerular filtration rate improves prediction of long-term all-cause mortality in older patients with chronic kidney disease. Rejuvenation Res, 15(1), 82-88.

Pilotto A, Sancarlo D, Franceschi M, Aucella F, D'Ambrosio P, Scarcelli C, Ferrucci L (2010) A multidimensional approach to the geriatric patient with chronic kidney disease. Journal of nephrology, 23, S5-10.

Pilotto A, Sancarlo D, Panza F, Paris F, D'Onofrio G, Cascavilla L, Addante F, Seripa D, Solfrizzi V, Dallapiccola B, Franceschi M, Ferrucci L (2009) The Multidimensional Prognostic Index (MPI), based on a comprehensive geriatric assessment predicts short- and long-term mortality in hospitalized older patients with dementia. J Alzheimers Dis, 18(1), 191- 199.

Pilotto A, Sancarlo D, Pellegrini F, Rengo F, Marchionni N, Volpato S, Ferrucci L, Group FSS (2016) The Multidimensional Prognostic Index predicts in-hospital length of stay in older patients: a multicentre prospective study. Age Ageing, 45(1), 90-96.

Pilotto A, Topinkova E, Michalkova H, Polidori MC, Cella A, Cruz-Jentoft A, von Arnim CAF, Azzini M, Gruner H, Castagna A, Cenderello G, Custureri R, Custodero C, Zieschang T, Padovani A, Sanchez-Garcia E, Veronese N, Investigators MCSG (2022) Can the Multidimensional Prognostic Index Improve the Identification of Older Hospitalized Patients with COVID-19 Likely to Benefit from Mechanical Ventilation? An Observational, Prospective, Multicenter Study. J Am Med Dir Assoc, 23(9), 1608.e1-1608.e8.

Pilotto A, Veronese N, Daragjati J, Cruz-Jentoft AJ, Polidori MC, Mattace-Raso F, Paccalin M, Topinkova E, Siri G, Greco A, Mangoni AA, Maggi S, Ferrucci L, Investigators MA (2019) Using the Multidimensional Prognostic Index to Predict Clinical Outcomes of Hospitalized Older Persons: A Prospective, Multicenter, International Study. J Gerontol A Biol Sci Med Sci, 74(10), 1643-1649.

Ritt M, Bollheimer LC, Sieber CC, Gassmann KG (2016) Prediction of one-year mortality by five different frailty instruments: A comparative study in hospitalized geriatric patients. Arch Gerontol Geriatr, 66, 66-72.

Ritt M, Radi K, Schwarz C, Bollheimer L, Sieber C, Gaßmann K (2016) A comparison of frailty indexes based on a comprehensive geriatric assessment for the prediction of adverse outcomes. The journal of nutrition, health & aging, 20, 760-767.

Rodriguez-Pascual C, Vilches-Moraga A, Paredes-Galan E, Ferrero-Marinez AI, Torrente- Carballido M, Rodriguez-Artalejo F (2012) Comprehensive geriatric assessment and hospital mortality among older adults with decompensated heart failure. Am Heart J, 164(5), 756-762.

Sancarlo D, D’Onofrio G, Franceschi M, Scarcelli C, Niro V, Addante F, Copetti M, Ferrucci L, Fontana L, Pilotto A (2011) Validation of a Modified-Multidimensional Prognostic Index (m-MPI) including the Mini Nutritional Assessment Short-Form (MNA-SF) for the prediction of one-year mortality in hospitalized elderly patients. The journal of nutrition, health & aging, 15, 169-173.

Sancarlo D, Pilotto A, Panza F, Copetti M, Longo MG, D'Ambrosio P, D'Onofrio G, Ferrucci L, Pilotto A (2012) A Multidimensional Prognostic Index (MPI) based on a comprehensive geriatric assessment predicts short- and long-term all-cause mortality in older hospitalized patients with transient ischemic attack. J Neurol, 259(4), 670-678.

Verholt AB, Gregersen M, Gonzalez-Bofill N, Hansen TK, Ebdrup L, Foss CH, Lietzen LW (2021) Clinical presentation and outcomes of COVID-19 in older hospitalised patients assessed by the record-based multidimensional prognostic index, a cross-sectional study. Eur Geriatr Med, 12(6), 1147-1157.

Volpato S, Bazzano S, Fontana A, Ferrucci L, Pilotto A, Group MPTS (2015) Multidimensional Prognostic Index predicts mortality and length of stay during hospitalization in the older patients: a multicenter prospective study. J Gerontol A Biol Sci Med Sci, 70(3), 325-331.

Zheng PP, Yao SM, Shi J, Wan YH, Guo D, Cui LL, Sun N, Wang H, Yang JF (2020) Prevalence and Prognostic Significance of Frailty in Gerontal Inpatients With Pre- clinical Heart Failure: A Subgroup Analysis of a Prospective Observational Cohort Study in China. Front Cardiovasc Med, 7, 607439.
